# *Cbf *genes of the Fr-A2 allele are differentially regulated between long-term cold acclimated crown tissue of freeze-resistant and – susceptible, winter wheat mutant lines

**DOI:** 10.1186/1471-2229-9-34

**Published:** 2009-03-23

**Authors:** Fedora Sutton, Ding-Geng Chen, Xijin Ge, Don Kenefick

**Affiliations:** 1 Plant Science Department, South Dakota State University, Plant Science Building, Jackrabbit lane, Brookings, SD 57007, USA; 2 Department of Mathematics and Statistics, South Dakota State University Brookings, SD 57007; Box 2220, Brookings, SD 57007, Office: Harding Hall 118, USA

## Abstract

**Background:**

In order to identify genes that might confer and maintain freeze resistance of winter wheat, a comparative transcriptome analysis was performed between control and 4 wk cold-acclimated crown tissue of two winter wheat lines that differ in field freeze survival. The lines, generated by azide mutagenesis of the winter wheat cultivar 'Winoka' were designated FR (75% survival) and FS (30% survival). Using two winter lines for this comparative analysis removed the influence of differential expression of the vernalization genes and allowed our study to focus on *Cbf *genes located within the *Fr-A2 *allele independent of the effect of the closely mapped *Vrn *allele.

**Results:**

Vernalization genes, (*Vrn-A1*, *B1 *and *D1*), and the transcription factor gene, *TaVrt-2*, were up-regulated to the same extent in FR and FS lines with cold acclimation thus confirming that azide mutagenesis had not modified the winter habitat of the lines. One category of *Cbf *genes, (*Cbf-2*, -*A22 *and B-22) reflected an increase in level of expression with cold acclimation in both FR and FS lines. Another category of *Cbf *genes (*Cbf-3*, -*5*, -*6*, -*12*, -*14 *and -*19*) were differentially expressed between cold-acclimated FR and FS lines relative to the non-acclimated controls. Comparison of expression patterns of the two categories of *Cbf *genes with the expression patterns of a set of ABA-dependent and -independent *Cor/Lea *genes revealed similar patterns of expression for this sample of *Cor/Lea *genes with that for *Cbf-2 *and *-22*. This pattern of expression was also exhibited by the *Vrn *genes.

**Conclusion:**

Some *Cor/Lea *genes may be co-regulated by the *Vrn *genes during cold acclimation and the *Vrn *genes may also control the expression of *Cbf-2, -A22 and -B22*. The increased freeze survival by the FR line and the increase in expression levels of wheat *Cbf *genes, *Cbf-3*, -*5*, -*6*, -*12*, -*14 *and -*19 *with cold acclimation in the FR line suggests a possible gain of function mutation resulting in higher levels of expression of these *Cbf *genes and increased freeze survival.

## Background

In order for winter cereals to switch from vegetative to reproductive phase, they must undergo a low temperature regime described as veranlization. Recessive alleles at the major *Vrn *loci (*vrn-A1, vrn-B1, vrnB4, vrn D1, vrn D5*) results in winter wheat and a dominant allele at one or more of these loci results in spring wheat [1-4]. The low non-freezing temperatures needed for vernalization are also needed for cold acclimation which is defined as the period of exposure to low non-freezing temperatures necessary for freeze survival.

Cold acclimation for wheat is usually 2–4°C for 4–6 wk (autumn) conditions. The ability to survive freezing temperatures has been represented by various terms including frost tolerance, winter hardiness, frost hardiness, freezing tolerance, freeze survival, cold resistance and freeze resistance. For consistency, we use the term freeze resistance as was done in our report on water loss by hard red winter wheat during cold acclimation [5]. The *Fr *loci which have been linked to freeze resistance have been mapped to all 3 genomes on the long arm of chromosome 5 in close approximation to the *Vrn *loci [6-8]. Therefore, attempts to identify genes linked to freeze resistance by comparing gene expression between winter and spring types have been confounded by the involvement of the *Vrn *loci which is also low-temperature regulated.

In an attempt to reduce the *Vrn *loci contribution to the study of freeze resistance in winter wheat, various near isogenic lines (NILs) for the *Vrn-1 *genes have been generated. These include: two *Vrn-1 *NILs developed from the sping cv. 'Triple Dirk' [9,10] and another set developed from the spring cv. Manitou (*Vrn-A1*) and the winter cv. Norstar (*vrn-A1*)[11,12]. In this study, in order to normalize for the involvement of the *Vrn *loci, we made use of two winter wheat mutant lines that vary in freezing survival [13]. We refer to the lines as freeze resistant (FR) or freeze susceptible (FS) based on their ability as a result of cold acclimation to withstand freezing temperatures in the fields (Table 1).

**Table 1:** Field freeze survival data.

**Entry**	**% Freeze Survival**	**Mean Height (cm)**
Winoka	35%	78
16029 (FR)	75%	60
16169 (FS)	30%	63

The percentage survival reported represents the average of two replications. The mean height for these lines were obtained from three replications at two locations in SD.

A significant body of research exists that describes the involvement of *Cor/Lea *genes during cold acclimation in cereals. A few of these include cold acclimation of barley and wheat [14-16]. We have contributed to these studies by examining the effect of cold acclimation on ABA-regulated barley genes such as *Hva1 *[17] and wheat genes not regulated by ABA such as *Tacr7 *[13]. Wheat ESTs are available for functional genomics [18] and include wheat *Cor/Lea *genes known to be controlled by an ABA-independent pathway: *Wcor15, Wcor14, Wcs19, Wlt10*, and *Wcs120 *[19,15,20] and others that respond to cold acclimation through an ABA-dependent pathway: *Wrab17, Wrab18, Wrab19 *and *Wcor825 *[21-24].

An understanding of the role of the *Cor/Lea *genes can be better achieved if the length of cold acclimation and the tissue used for cold acclimation is defined more carefully. Ganeshan *et al *[12] have demonstrated that *Wcor14 *levels in leaf tissue of winter Norstar declined by 21 d of cold acclimation, whereas *Wcor14 *levels were sustained to 63 d cold acclimation of crown tissue. Knox *et al *[25] reported no differential expression with 12°C treatment of crown tissue from wheat lines varying in freeze resistance. In our study, we were particularly interested in understanding sustained 4°C cold acclimation for at least 4 wk (the conditions we have determined distinguish FR from FS lines based on freeze survival) and specifically we wished to understand the gene expression levels in the crown tissue, the most freeze resistant part of the plant [26,27].

With the identification of many *Cor/Lea *genes, researchers concluded that a better understanding of the role of such genes in freeze resistance could be ascertained by determining how these genes are regulated at the transcriptional level. Such studies led to the discovery of C-repeat/dehydration-responsive elements (CRT/DRE) in the promoter region of the *Cor/Lea *genes[28,29] and the identification of the proteins that bind the CRT/DRE as CRT-binding factor/DRE-binding proteins 1 (CBF/DREB1) [30,31]. The involvement of CBFs in the cold acclimation process has been described [32-35].

Vagujfalvi *et al*., [36,37] using *Cbf *gene specific primers examined the response of *Cbf *genes in *T. aestivum*. They reported that a 2 hr, 2°C (LT) treatment caused a four-fold induction of genes *TaCbf-14, -15 *and *-16 *in frost tolerant lines harboring the frost tolerant allele CNN *Fr-A2*, compared to the frost sensitive lines. Stockinger *et al *[38] studying *Cbf *gene expression in barley reported that *HvCbf-2 *and *-4 *were expressed to higher levels in frost tolerant lines harboring the 'Nure' *Fr-H2 *allele than in the frost sensitive 'Tremois' *Fr-H2 *allele. The wheat species *T. aestivum *and *T. monococcum *have been reported to contain around ten *Cbf *groups of which groups *CbfIIId, IVa, IVb, IVc *and *IVd *were induced to higher levels in the winter cultivar in response to LT [39]. These researchers postulated that the higher inherited and LT inducible *Cbf *expression may mean that these *Cbf *groups are major components for control of wheat to develop freezing resistance.

Miller *et al *[40] generated seven recombination events within the C*bf *cluster located in the *Fr-A2 *allele by crossing *T. monococcum *genotypes DV92 (spring frost sensitive) with G3116 (winter frost tolerant). These recombination events were studied by Knox *et al *[25] who described three gene clusters at the *Fr-A2 *locus of *T. monococcum*. These were designated: (1) Proximal (*Cbf-2, -4, -9 and -17); *(2) Central (*Cbf-14, -15, -12*) and (3) Distal (*Cbf-16, -13, -3 and -10*). From the frost tolerance data generated by Knox *et al.*,[25], the locus for frost tolerance was completely linked to the central gene cluster (*Cbf-14, -15, -12*).

We used the Affymetrix Wheat chip for comparative transcriptome analyses between an FR and an FS line with RNA from non-acclimated and long-term cold acclimated (4 wk) winter wheat crown tissue. In this report we highlight the behavior of the *Cbf *genes of the *Fr-A2 *loci and propose a model for their involvement in confering freeze resistance.

## Results

### Comparative transcriptome profiling

Transcript levels were averaged for two technical replicates each consisting of three pooled biological replicates. FR represents the freeze resistant line (75% field survival) and FS represents the freeze susceptible line (30% field survival). Heat maps are presented in the respective section. The colors red and green are used to indicate up-regulation and down-regulation respectively in response to cold acclimation relative to non-acclimated control samples. The black color represents no change in gene expression in response to cold acclimation. The microarray data has been deposited at the Plant Expression database  accession number TA22. The data has also been deposited in the GEO database, Accession # = GSE14697 .

### Winter habitat mutant lines

**Figure 1:**
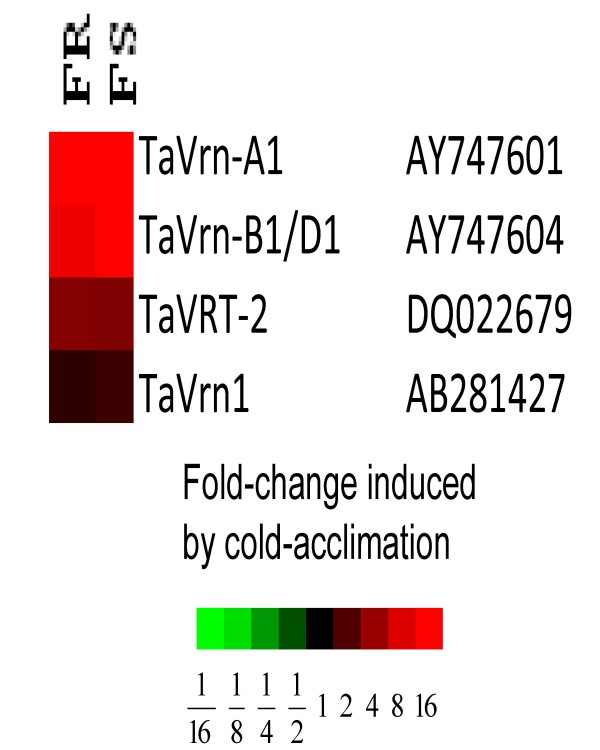
**Expression of Vernalization genes in two mutant winter wheat lines**. Values are represented as a heatmap of fold change induced by cold acclimation in FR (freeze resistant, 75% field survival) compared with FS (freeze susceptible, 30% field survival) lines relative to control non-acclimated samples. Colours used to represent fold-change in gene expression are: red – up-regulated and black – not regulated. The accession numbers are listed next to the gene names.

A distinction between winter and spring habitat can be made based on the expression of various *Vrn *genes, (*Vrn-A1, -B1*, -*D1*), and the genes for the vernalization transcription factors *TaVrn1/TaVrt-1 *and *TaVrt-2 *in response to cold acclimation. The transcriptome analyses data for these five genes are depicted in Figure 1. Genes, (*Vrn-A1, -B1*, and -D1), showed greater than 8-fold regulation to the same extent in FR as FS with cold acclimation relative to the control non-acclimated samples. *TaVrt-2 *was expressed to 4-fold levels in both FR and FS. *TaVrn1 *was not induced with cold acclimation in either FR or FS lines.

### *Cbf *genes expression patterns

**Figure 2:**
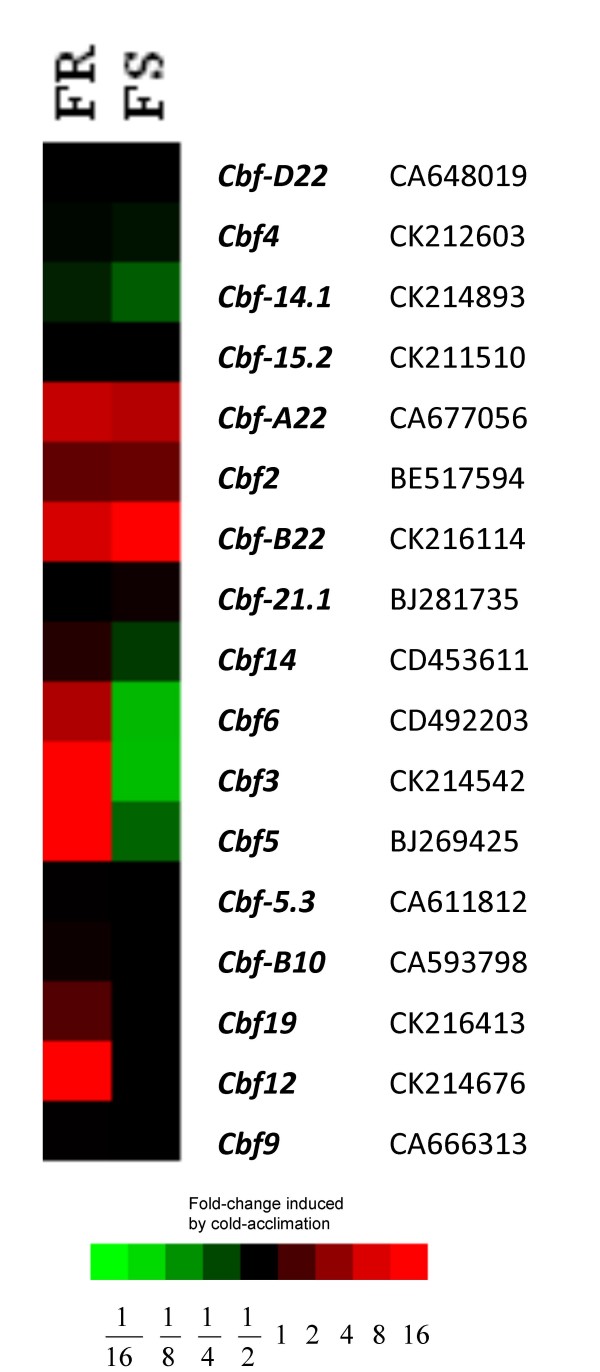
**Expression of *Cbf *genes in two mutant winter wheat lines**. Values are represented as a heatmap of fold change induced by cold acclimation relative to control or non-acclimation in FR (freeze resistant, 75% field survival) compared with FS (freeze susceptible, 30% field survival) lines. Colours used to represent fold-change in gene expression are: red – up regulated, green – down regulated and black – not regulated. The accession numbers are listed next to the gene names.

As seen in Figure 2, transcript levels for 17 *Cbfs *identified on the chip appeared to fall into three basic categories. Category I: Those with very low levels of expression in the crown tissue of both FR and FS lines with and without cold-acclimation (*Cbf-D22, -4, -15.2, -21.1, -5.3*, -*10 *and *-9*). Category II: *Cbfs *that are up-regulated with cold acclimation in both FR and FS lines (*Cbf-A22, – B22*, and *-2*). Category III: *Cbf *genes that are upregulated in cold-acclimated FR crown tissue but not in cold-acclimated FS crown tissue (*Cbf-14, -6, -3, -5*, -*19 *and -*12*). All expression levels are presented as relative to the control non-acclimated samples.

### *Cor/Lea *genes expression patterns

**Figure 3:**
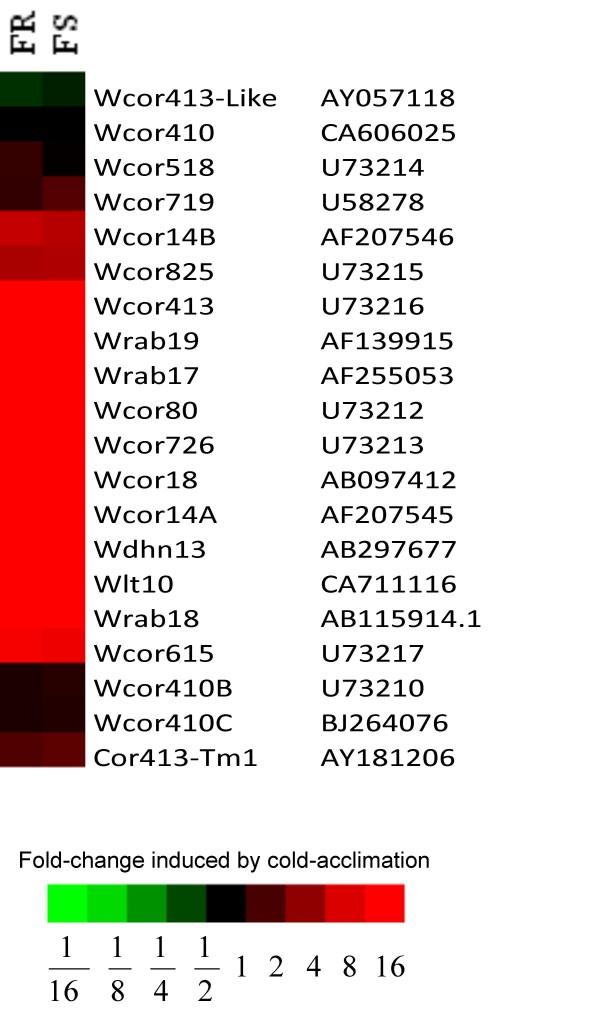
**Expression of *Cor/Lea *genes in two mutant winter wheat lines**. A heatmap is used to represent the fold change induced by cold acclimation in FR (freeze resistant, 75% field survival) compared with FS (freeze susceptible, 30% field survival) lines of a sample of *Cor/Lea *genes. Colours used to represent fold-change in gene expression are: red – up regulated, green – down regulated and black – not regulated. The accession numbers are listed next to the gene names.

Transcription levels of 20 *Cor/Lea *genes including ABA-dependent (*Wrab-17, -18, -19 *and *Wcor825*) and ABA-independent (*Wcor-14A,-*14*B*, and *Wlt10) Cor *genes are depicted in Figure 3. Relative to the non-acclimated control samples, these *Cor/Lea *genes were up-regulated in FR and FS cold-acclimated crown tissue and there was also no significant difference between the pattern of expression of the ABA -dependent and -independent genes sampled.

### Expression analyses by real-time qRT-PCR

**Figure 4:**
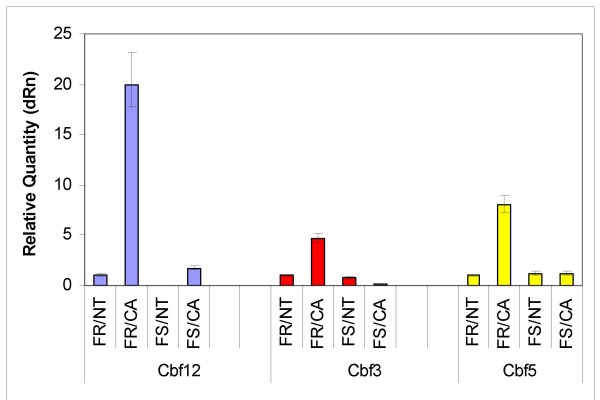
**Confirmation of microarray expression pattern by RT-PCR**. Total RNA isolated from crown tissue of FR and FS winter wheat lines and primers (Table 2) were used to determine *Cbf-3, -5 *and *-12 *expression levels. Values were calculated with the MXPro-Mx300P Stratagene software with the 18S levels as normalizers and the FR/NT levels as the calibrator. The relative quantity of gene expression for NT = untreated or non-treated samples, CA = cold-acclimated samples of FR and FS lines are depicted.

The pattern of gene expression from the microarray was verified by examining the relative transcript levels for candidate *Cbf *genes normalized to the 18S ribosomal transcript levels by real time qRT-PCR. Since our criterion for relatedness to freeze resistance is based on differential expression between FR and FS lines, we present RT-PCR data on members of the *Cbfs *that were differentially regulated. As seen in Figure 2., the high level expressing *Cbf *genes of this group are *Cbf-6, -3, -5, and -12*. Since *Cbf-6 *and *-3 *are very similar but different from *Cbf-5 *and *-12*, wechose to examine by RT-PCR *Cbf-*3, -5 and -12. The results are depicted in Figure 4. As viewed by relative quantity (Figure 4) all three genes were not significantly expressed in control or non-acclimated FR and FS tissue. However all three were upregulated in response to 4 wk cold acclimation of FR crown tissue but not significantly changed from baseline expression in 4 wk cold-acclimated FS crown tissue. The pattern of expression was similar to that observed from the microarray.

## Discussion

### Normalization of vernalization requirement

We believe that studies to unravel the basis of freeze resistance in winter wheat are confounded by the use of comparisons between winter and spring cultivars. Therefore, we designed our experiments to normalize for the contribution from the Vrn loci by using two winter types derived from the same starting winter cultivar. Our comparison of Vrn gene expression between FR and FS with cold acclimation (Figure 1) leads us to conclude that the mutagenesis procedure used to generate these lines did not affect the molecular basis of the winter habitat.

### Expression of *Cbf *genes at the *Fr *loci between FR and FS lines

Having verified that the *Vrn *genes were being expressed to the same levels in FR and FS lines in response to cold acclimation, we could now examine the expression of the *Cbf *gene clusters described by Badawi *et *al [39] and Knox *et al *[25]. In particular we queried the *Cbf *genes that were available on the Affymetrix wheat chip that had been mapped to the long arm of *T. aestivum *chromosome 5. According to Badawi *et al *[39] the *Cbf *genes at the *Fr *loci included *Cbf-2*, -*3, -4, -6, -9, -10, -12, -14, -15, -19, -20*, -*21 *and -22. However, according to Knox *et al*., [25] the *Cbfs *at the *Fr *loci are *Cbf-2,-3, -4, -9,-10, -12,-14 *and -*15*. These researchers identified *Cbf-6, -19, -20, -21 *and *-22 *as not belonging to the frost tolerance gene clusters. Our data leads us to agree partially with Knox *et al*., [25]. Genes not differentially expressed between FR and FS lines in our system are assumed to be not associated with freeze resistance. Thus like Knox *et al*., [25] we place *Cbf-21 *and *-22 *as unassociated with winter wheat freeze resistance. However we disagree with the findings of Knox *et al*., [25] relative to *Cbf-6 *and *-19*. In our system, the *Cbf *genes differentially regulated between FR and FS lines included *Cbf-6 *and *-19*. These were described by Knox *et al*., [25] as belonging to the unlinked cluster. We also observed differential expression for genes from the central (*Cbf12, 14*) and distal (*Cbf3*) clusters on the long arm of chromosome 5 (Figure 2). Additionally *Cbf5 *was identified as differentially regulated between FR and FS (Figure 2 &4). However, it is not located on the long arm of chromosome 5. Thus, the relationship of *Cbf5 *to the other *Cbf *genes that map to the *Fr *loci will be of interest in determining the role of *Cbf*s in other plant responses other than freeze survival or the identification of other loci that associate with the *Fr *loci to confer freeze resistance.

### *Cbf*, *Cor/Lea *gene expression pattern comparisons in FR and FS lines with cold acclimation

From expression data, it is possible to cluster genes that are co-expressed in response to a stimulus. Co-expression usually suggests coordinated gene transcription and allows for the identification of transcription factors and the genes that they regulate. Comparisons of co-expression have been performed to identify transcription factors [41] and biosynthetic enzymes [42]. These findings led us to attempt to identify which of the *Cor/Lea *genes are controlled by which *Cbfs *by comparing the expression patterns of the *Cbfs *with the *Cor/Lea *expression patterns. If a similar pattern of expression is an indicator of coordinated gene transcription then we can deduce from our results that the sample of *Cor/Lea *genes examined that belong to both the ABA-dependent and ABA-independent pathways are coordinately regulated (Figure 3). And since the pattern of expression of these *Cor/Lea *genes is similar to that of *Cbf-2 *and -*22 *then we can assume that *Cbf-2 *and -*22 *encode the CBFs that control the expression of this cluster of *Cor/Lea *genes.

### Relationship between *Vrn *genes and *Cor/Lea *genes

Another set of genes with similar expression patterns with the *Cor/Lea *genes were the *Vrn *genes (Figure 1). This co-expression pattern suggests that the *Cor/Lea *up-regulation may be via the *Vrn *genes signaling pathways. This hypothesis is supported by the findings of Danyluk et al., [43] that the *Vrn-A1/vrn-A1 *locus regulates *Cor/Lea *gene induction. We propose that this sample of *Cor/Lea *genes are regulated by *Vrn-A1,-B1 *and -*D1 *genes during cold acclimation. It will be of value to determine the relationship between the coordinated expression of the *Cbf- *gene set (*Cbf-2, -A22*, and *-B22*) and the *Vrn - *gene set (*Vrn-A1,-B1 *and -*D1*). The differential *Cbf *gene expression levels observed for *Cbf-3, -5, -6, -12 -14 *and *-19 *did not correlate with gene expression patterns for any of the highly expressed Cor genes examined. Thus we can assume that at 4 wk cold acclimation these *Cbf *genes were not involved in regulation of this group of *Cor *genes.

### Relationship between cold acclimation-induced expression of *Cbf *and freeze resistance of FR and FS lines

**Figure 5:**
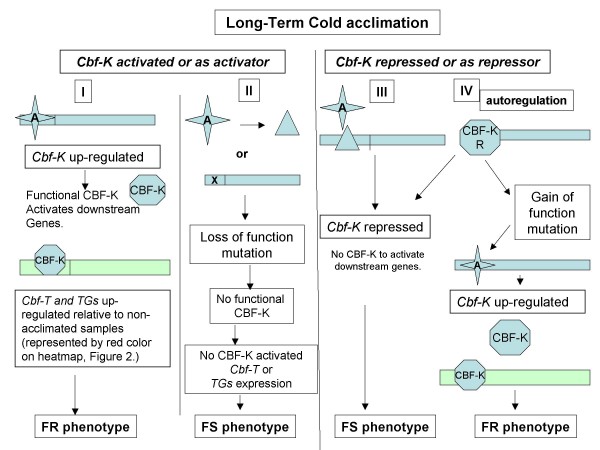
**Model Relating Cold-Acclimation, *Cbf-*genes and FR versus FS Freeze Resistance**. From the central cluster, we choose *Cbf-12 *or *-14 *as a key regulator (Cbf-K) of target *Cbf *genes such as *Cbf-3, -5 *and *-6 *(*Cbf-T*) as well as down-stream target genes (TGs). (I) Activator (A) is able to upregulate *Cbf-K *resulting in a functional CBF-K and activation of downstream events. (II) Modification of activator (A) by mutagenesis or a mutation in the activator binding site could result in loss of downstream actions and the lower level of freeze survival of the FS line. (III) *Cbf-K *may be repressed by an external repressor of by repressor function of *CBF-K*. As a result the downstream activities are not operational and the FS line is observed. (IV) A mutation resulting in disruption of repression of *Cbf-K *would lead to up regulation of *Cbf-T *and its *TGs *and the increased freeze survival of the FR line.

To unravel the complex inter-relationship between the *Cbf *genes, cold acclimation and freeze resistance we propose the following model. We propose that one of the *Cbf *genes belonging to the central cluster (*Cbf-12 *or *-14*) may serve as a key regulator of other genes including other *Cbf *genes. For simplicity we designate that key regulator as *Cbf-*K and we designate the target *Cbf *genes as *Cbf-*T. Possible *Cbf-Ts *would include *Cbf-3, -5 *and *-6 *which then control expression of down-stream target genes designated *TGs*. We propose that with cold acclimation, *Cbf-K *can be activated resulting in the production of functional CBF-K which activates downstream genes to produce the FR phenotype (Figure 5I). The FS phenotype could be generated from the FR phenotype by a "loss of function" mutation as a result of mutagenesis. This could be achieved if the *Cbf-K *activator binding site is mutated resulting in low-level to no increase in transcription of the *Cbf-K *gene (Figure 5II).

However, because wheat is hexaploid, and the FR line has a significant increase in survival compared to the WT or the FS line (Table 1.), we believe that we are observing the effects of a "gain of function" mutation. It is possible that in FS lines, Cbf *-K *is repressed by an external factor or by autoregulation and as a result *Cbf-T *and its *TGs *are not activated (Figure 5III). The azide mutagenesis could have resulted in either a mutation in the repressor binding site for *Cbf-K *or a mutation to the repressor domain, allowing for an active CBF-K and therefore activation of the downstream genes that lead to the freeze survival properties expressed by FR lines (Figure 5IV).

Future studies will allow us to determine whether *Cbf-12 *and/or -*14 *play a key role in conferring freeze resistance by any of the possible mechanisms proposed and whether *Cbf-3, -5, -6 *and *-19 *are target genes or master switches.

## Conclusion

The FR and FS lines used in this study will serve as valuable tools for analayzing freeze resistance of HRWW and genes differentially expressed between FR and FS lines. The *Cbf genes 3, 5,6, 12, 14 *and *19 *are potential markers for freeze survival of HRWW.

## Methods

### Generation of FR and FS 'Winoka' mutant lines

'Winoka' is a release from the South Dakota Experiment station [45]. This release was used in an azide mutagenesis project in 1983. The sodium azide mutagenesis procedure was followed [46]. Certified Winoka seeds (1000) were imbibed in iced-water for 16 h followed by continued imbibition at 20°C for 8 hr. The seeds were then transferred to 1 mM Sodium azide in 0.1 M potassium phosphate buffer (pH 3) for 2 hr at 20°C. The 20°C azide-treatments were performed with vigorous bubbling of air. At the end of the treatment, the seeds were rinsed for 30 min in tap water and then air-dried for 3 d. In preparation for planting, the seeds were hydrated for 24 hr at 25°C before vernalization for 6 wk. After vernalization the seeds were germinated for 3 d and viable M1 seedlings (656) were obtained. From this population of M1 Winoka mutagenized plants, 20 were identified as potential mutants based on characteristics such as striated leaves, height, varied heading date or loss of plant vigor. Several of these lines were tested for freeze survival at various locations in SD and ND. Two of these lines that varied in freeze survival were chosen for further study. An average of 2 replications of M5 lines in the Northern Uniform Winter Hardiness Nursery at Casselton, North Dakota in 1988 revealed that SD16029 had 75% survival and was designated as freeze resistant (FR), SD16169 was designated freeze susceptible (FS) with 30% freeze survival (Table 1).

### Plant Material and Growth Conditions

FR and FS plants (4 plants/pot) were grown in the green house at 22–28°C with 14 h photoperiod. Soil water was maintained at 0.3 – 0.44 kg H_2_O kg^-1 ^and plants transferred to 4°C cold room for cold acclimation after reaching the fourth leaf stage as described [5]. Since crown tissue is known to be the most freeze resistant part of the plant [47,27], this was the tissue of choice for our study. The crown was defined as the non-photosynthetic region located just above the roots. We avoided any associated leaf or root material. At the 3^rd ^to 4^th ^leaf stage, control/non-treated (NT) plants were harvested, the crowns excised with a clean scalpel into liquid N_2 _and stored at -80°C for RNA isolation. Treated or cold acclimated (CA) crown samples were derived from plants that at the 3^rd ^to 4^th ^leaf stage were transferred from the green house directly to a 4°C chamber for 4 wk. At the end of cold acclimation, while maintaining the temperature at 4°C, the crown tissue was removed from each plant and the tissue immediately frozen in liq N_2 _and stored at -80°C.

### RNA isolation

Frozen crown tissue from each of three independent replicates for FR and FS ctrl/NT samples and three independent replicates for FR and FS cold-acclimated (CA) plants were used for RNA isolation. The crowns for each of the three replicates were pooled and total RNA isolated by a modification of the procedure by Chirgwin *et al.*, [48] as described by Han [49]. Frozen powdered tissue was resuspended and homogenized (1 g/3–5 ml) in 4 M GSCN buffer (4 M GSCN, 50 mM NaOAc (pH 5.2), 10 mM EDTA (pH 8.0), 7% (v/v) 2-mercaptoethanol). SDS (10%) was added to a final concentration of 0.1%. The homogenate was centrifuged at 12,000 g for 10 min. The resulting supernatant was filtered through two layers autoclaved Mira cloth to remove any loose debris. The cleared homogenate was then loaded on a 9 ml CsCl cushion (5.7 M CsCl, 0.1 M EDTA (pH 7.5)) centrifuged at 113,000 g for 26 h at 20°C in a SW28 rotor. The supernatant was discarded and the RNA pellet resuspended in HE/SDS (10 mM Hepes (ph 7.4), 1 mM EDTA, 0.1% SDS). RNA was reprecipitated out of solution with standard NaOAc/ethanol procedure. Some samples were resuspended in HE buffer and others in 100% formamide for long-term storage.

### Transcription Profiling

Prior to submission for use in microarry analyses, RNA stored in formamide were reprecipitated with standard NaOAc/ethanol procedure, resuspended in depc-treated H_2_O. All samples were treated with DNase to remove any possible contaminating genomic DNA using the protocol as described for the Qiagen RNeasy kit.

The RNA samples were shipped on dry ice to UC Riverside and cDNA synthesis and hybridization performed with the affymetrix wheat chip as described 

### Data Analysis

Eight samples with 61,290 probe-sets from each sample were analyzed by R/Bioconductor [50,51]. Specifically, Robust Multi-Array Average with help of probe sequence information, which is known as gcRMA [52-54] was used for microarray background correction and normalization. Gene expression data for selected *Cbf *and *Cor *genes were analyzed and visualized by the Cluster and Treeview software [55].

### Real-time RT-PCR

Aliquots of the RNA used for the microarray analyses were maintained at -80°C until needed for RT-PCR verification of microarray data. RNA was quantified and examined for quality. cDNA was synthesized with total RNA using the cDNA synthesis kit according to manufacturer's directions (Applied Biosystems). Using the probe ID target sequences from the Affymetrix website, primers were designed to allow for real-time amplification of the chosen *Cbf *genes. Table 2. contains a list of the primers used in the amplification. Stratagene SYBR Green PCR master mix or Applied Biosystems qRT-PCR master mixed was used for RT-PCR or PCR as needed. The Stratagene real-time equipment located in the SDSU functional genomics core was used with the MxPro-Mx3000P software to collect and analyze the data. The data collected with the SYBR Green with dissociation curve program was converted to the Comparative PCR format. FR/NT RNA was used as the calibrator and samples with the 18S primers were used as the normalizer. Dyes used included SYBR Green and Rox reference dye.

**Table 2:** Primers used for RT-PCR.

Gene	Forward Primer	Reverse Primer
C*bf3*	AATCGTCGTCTGAGTCTGACAGTG	TTCCGGGAACAAGTCAAGCCT
*Cbf5*	ATGAGCCGACGACGACTGCAAC	TACGCTGAGTGATGTTGTACGGCAG
*Cbf12*	AAATGGACGCGGGCACGTACTA	TCATCAGTGGTTCCATAGCGCC

## Authors' contributions

FS and DK contributed to the experimental design, plant physiology, abiotic stress treatments and molecular analyses. FS performed the real time PCR, analyzed the data and drafted the manuscript. DGC and XG contributed to the experimental design and the bioinformatic analyses. All authors read and approved the final manuscript.

## References

[B1] Law CN (1966). The Location of Genetic Factors Affecting a Quantitative Character in Wheat. Genetics.

[B2] Pugsley A (1971). A genetic analysis of the spring-winter habit of growth in wheat. Aust J Agric Res.

[B3] Stelmakh A (1993). Genetic effects of *Vrn *genes on heading date and agronomic traits in bread wheat. Euphytica.

[B4] Košner J, Pánková K (1998). The detection of allelic variants at the recessive loci of winter wheat. Euphytica.

[B5] Kenefick DG, Koepke JA, Sutton F (2002). Plant water uptake by hard red winter wheat (Triticum aestivum L.) genotypes at 2 degrees C and low light intensity. BMC Plant Biol.

[B6] Galiba G, Quarrie SA, Sutka J, Morgounov A, Snape JW (1995). RFLP mapping of the vernalization (*Vrn1*) and frost resistance (*Fr1*) genes on chromosome 5A of wheat. Theor Appl Genet.

[B7] Snape JW, Semikhodskii A, Fish L, Sarma RN, Quarrie SA, Galiba G, Sutka J (1997). Mapping frost tolerance loci in wheat and comparative mapping with other cereals. Acta Agric Hung.

[B8] Toth B, Galiba G, Feher E, Sutka J, Snape JW (2003). Mapping genes affecting flowering time and frost resistance on chromosome 5B of wheat. Theor Appl Genet.

[B9] Barrett B, Bayram M, Kidwell K, Weber WE (2002). Identifying AFLP and microsatellite markers for vernalization response gene *Vrn- *B1 in hexaploid wheat using reciprocal mapping populations. Plant Breeding.

[B10] Kobayashi F, Takumi S, Kume S, Ishibashi M, Ohno R, Murai K, Nakamura C (2005). Regulation by Vrn-1/Fr-1 chromosomal intervals of CBF-mediated Cor/Lea gene expression and freezing tolerance in common wheat. J Exp Bot.

[B11] Limin AE, Fowler DB (2002). Developmental traits affecting low-temperature tolerance response in near-isogenic lines for the Vernalization locus Vrn-A1 in wheat (Triticum aestivum L. em Thell). Ann Bot (Lond).

[B12] Ganeshan S, Vitamvas P, Fowler DB, Chibbar RN (2008). Quantitative expression analysis of selected COR genes reveals their differential expression in leaf and crown tissues of wheat (Triticum aestivum L.) during an extended low temperature acclimation regimen. J Exp Bot.

[B13] Gana JA, Sutton F, Kenefick DG (1997). cDNA structure and expression patterns of a low-temperature-specific wheat gene tacr7. Plant Mol Biol.

[B14] Cattivelli L, Bartels D (1990). Molecular cloning and characterization of cold-regulated genes in barley. Plant Physiol.

[B15] Ohno R, Takumi S, Nakamura C (2001). Expression of a cold-responsive Lt-Cor gene and development of freezing tolerance during cold acclimation in wheat (Triticum aestivum L.). J Exp Bot.

[B16] Pearce R, Houlston CE, Atherton KM, Rixon JE, Harrison P, Hughes MA, Dunn MA (1998). Localization of expression of three cold-induced genes, blt101, blt4.9, and blt14, in different tissues of the crown and developing leaves of cold-acclimated cultivated barley. Plant Physiology.

[B17] Sutton F, Ding X, Kenefick DG (1992). Group 3 LEA Gene HVA1 Regulation by Cold Acclimation and Deacclimation in Two Barley Cultivars with Varying Freeze Resistance. Plant Physiol.

[B18] Houde M, Belcaid M, Ouellet F, Danyluk J, Monroy AF, Dryanova A, Gulick P, Bergeron A, Laroche A, Links MG (2006). Wheat EST resources for functional genomics of abiotic stress. BMC Genomics.

[B19] Tsvetanov S, Ohno R, Tsuda K, Takumi S, Mori N, Atanassov A, Nakamura C (2000). A cold-responsive wheat (Triticum aestivum L.) gene wcor14 identified in a winter-hardy cultivar 'Mironovska 808'. Genes Genet Syst.

[B20] Dong N, Danyluk J, Wilson KE, Pocock T, Huner NP, Sarhan F (2002). Cold-regulated cereal chloroplast late embryogenesis abundant-like proteins. Molecular characterization and functional analyses. Plant Physiol.

[B21] Tsuda K, Tsvetanov S, Takumi S, Mori N, Atanassov A, Nakamura C (2000). New members of a cold-responsive group-3 Lea/Rab-related Cor gene family from common wheat (Triticum aestivum L.). Genes Genet Syst.

[B22] Borovskii GB, Stupnikova IV, Antipina AI, Vladimirova SV, Voinikov VK (2002). Accumulation of dehydrin-like proteins in the mitochondria of cereals in response to cold, freezing, drought and ABA treatment. BMC Plant Biol.

[B23] Allagulova CR, Gimalov FR, Shakirova FM, Vakhitov VA (2003). The plant dehydrins: structure and putative functions. Biochemistry (Mosc).

[B24] Kobayashi F, Takumi S, Nakamura C (2008). Increased freezing tolerance in an ABA-hypersensitive mutant of common wheat. J Plant Physiol.

[B25] Knox AK, Li C, Vagujfalvi A, Galiba G, Stockinger EJ, Dubcovsky J (2008). Identification of candidate CBF genes for the frost tolerance locus Fr-Am2 in Triticum monococcum. Plant Mol Biol.

[B26] Olien C (1967). Freezing stresses and survival. Ann Rev Plant Physiol.

[B27] Chen TH, Gusta LV, Fowler B (1983). Freezing injury and root development in winter cereals. Plant Physiol.

[B28] Yamaguchi-Shinozaki K, Shinozaki K (1994). A novel cis-acting element in an Arabidopsis gene is involved in responsiveness to drought, low-temperature, or high-salt stress. Plant Cell.

[B29] Baker SS, Wilhelm KS, Thomashow MF (1994). The 5'-region of Arabidopsis thaliana cor15a has cis-acting elements that confer cold-, drought- and ABA-regulated gene expression. Plant Mol Biol.

[B30] Stockinger EJ, Gilmour SJ, Thomashow MF (1997). Arabidopsis thaliana CBF1 encodes an AP2 domain-containing transcriptional activator that binds to the C-repeat/DRE, a cis-acting DNA regulatory element that stimulates transcription in response to low temperature and water deficit. Proc Natl Acad Sci USA.

[B31] Liu Q, Kasuga M, Sakuma Y, Abe H, Miura S, Yamaguchi-Shinozaki K, Shinozaki K (1998). Two transcription factors, DREB1 and DREB2, with an EREBP/AP2 DNA binding domain separate two cellular signal transduction pathways in drought- and low-temperature-responsive gene expression, respectively, in Arabidopsis. Plant Cell.

[B32] Chen JQ, Dong Y, Wang YJ, Liu Q, Zhang JS, Chen SY (2003). An AP2/EREBP-type transcription-factor gene from rice is cold-inducible and encodes a nuclear-localized protein. Theor Appl Genet.

[B33] Gilmour SJ, Zarka DG, Stockinger EJ, Salazar MP, Houghton JM, Thomashow MF (1998). Low temperature regulation of the Arabidopsis CBF family of AP2 transcriptional activators as an early step in cold-induced COR gene expression. Plant J.

[B34] Shinozaki K, Yamaguchi-Shinozaki K (2000). Molecular responses to dehydration and low temperature: differences and cross-talk between two stress signaling pathways. Curr Opin Plant Biol.

[B35] Thomashow MF (2001). So what's new in the field of plant cold acclimation? Lots!. Plant Physiol.

[B36] Vagujfalvi A, Galiba G, Cattivelli L, Dubcovsky J (2003). The cold-regulated transcriptional activator Cbf3 is linked to the frost-tolerance locus Fr-A2 on wheat chromosome 5A. Mol Genet Genomics.

[B37] Vágújfalvi A, Aprile A, Miller A, Dubcovsky J, Delugu G, Galiba G, Cattivelli L (2005). The expression of several Cbf genes at the Fr-A2 locus is linked to frost resistance in wheat. Mol Genet Genomics.

[B38] Stockinger EJ, Skinner JS, Gardner KG, Francia E, Pecchioni N (2007). Expression levels of barley Cbf genes at the Frost resistance-H2 locus are dependent upon alleles at Fr-H1 and Fr-H2. Plant J.

[B39] Badawi M, Danyluk J, Boucho B, Houde M, Sarhan F (2007). The CBF gene family in hexaploid wheat and its relationship to the phylogenetic complexity of cereal CBFs. Mol Genet Genomics.

[B40] Miller AK, Galiba G, Dubcovsky J (2006). A cluster of 11 CBF transcription factors is located at the frost tolerance locus Fr-Am2 in Triticum monococcum. Mol Genet Genomics.

[B41] Hirai MY, Sugiyama K, Sawada Y, Tohge T, Obayashi T, Suzuki A, Araki R, Sakurai N, Suzuki H, Aoki K (2007). Omics-based identification of Arabidopsis Myb transcription factors regulating aliphatic glucosinolate biosynthesis. Proc Natl Acad Sci USA.

[B42] Persson S, Wei H, Milne J, Page GP, Somerville CR (2005). Identification of genes required for cellulose synthesis by regression analysis of public microarray data sets. Proc Natl Acad Sci USA.

[B43] Danyluk J, Kane NA, Breton G, Limin AE, Fowler DB, Sarhan F (2003). TaVRT-1, a putative transcription factor associated with vegetative to reproductive transition in cereals. Plant Physiol.

[B44] Danyluk J, Houde M, Rassart E, Sarhan F (1994). Differential expression of a gene encoding an acidic dehydrin in chilling sensitive and freezing tolerant gramineae species. FEBS Lett.

[B45] Wells DG, Lay CL, Buchenau GW, Johnson VA, Finney KF (1969). Registration of Winoka wheat. Crop Science.

[B46] Awan MA, Konzak CF, Rutger JN, Nilan RA (1980). Mutagenic Effects of Sodium Azide in Rice. Crop Science.

[B47] Gusta LV, Weiser CJ (1972). Nucleic Acid and Protein Changes in Relation to Cold Acclimation and Freezing Injury of Korean Boxwood Leaves. Plant Physiol.

[B48] Chirgwin JM, Przybyla AE, MacDonald RJ, Rutter WJ (1979). Isolation of biologically active ribonucleic acid from sources enriched in ribonuclease. Biochemistry.

[B49] Han K (1997). Partial cDNA of freeze resistance-related gene in wheat isolated by differential display. Master of Science Thesis.

[B50] Dudoit S, Fridlyand J (2002). A prediction-based resampling method for estimating the number of clusters in a dataset. Genome Biol.

[B51] Gentleman RC, Carey VJ, Bates DM, Bolstad B, Dettling M, Dudoit S, Ellis B, Gautier L, Ge Y, Gentry J (2004). Bioconductor: open software development for computational biology and bioinformatics. Genome Biol.

[B52] Irizarry RA, Bolstad BM, Collin F, Cope LM, Hobbs B, Speed TP (2003). Summaries of Affymetrix GeneChip probe level data. Nucleic Acids Res.

[B53] Irizarry RA, Hobbs B, Collin F, Beazer-Barclay YD, Antonellis KJ, Scherf U, Speed TP (2003). Exploration, normalization, and summaries of high density oligonucleotide array probe level data. Biostatistics.

[B54] Irizarry RA, Ooi SL, Wu Z, Boeke JD (2003). Use of mixture models in a microarray-based screening procedure for detecting differentially represented yeast mutants. Stat Appl Genet Mol Biol.

[B55] Eisen MB, Spellman PT, Brown PO, Botstein D (1998). Cluster analysis and display of genome-wide expression patterns. Proc Natl Acad Sci USA.

